# Clinical Course and Outcomes among COVID-19 Patients at the Hospitel in Bangkok: A Retrospective Study

**DOI:** 10.3390/tropicalmed7090238

**Published:** 2022-09-10

**Authors:** Jackrapong Bruminhent, Yosapan Kaewsanga, Werapoj Jiraaumpornpat, Vanlapa Arnuntasupakul, Thitiporn Suwatanapongched, Sasisopin Kiertiburanakul

**Affiliations:** 1 Division of Infectious Diseases, Department of Medicine, Faculty of Medicine Ramathibodi Hospital, Mahidol University, Bangkok 10400, Thailand; 2 Faculty of Medicine Ramathibodi Hospital, Mahidol University, Bangkok 10400, Thailand; 3 Department of Anesthesiology, Faculty of Medicine Ramathibodi Hospital, Mahidol University, Bangkok 10400, Thailand; 4 Division of Diagnostic Radiology, Department of Diagnostic and Therapeutic Radiology, Faculty of Medicine Ramathibodi Hospital, Mahidol University, Bangkok 10400, Thailand

**Keywords:** pneumonia, RT-PCR, SARS-CoV-2, corticosteroid, favipiravir

## Abstract

A hospitel is a hotel that has been designated as an extension of the healthcare facilities during the COVID-19 pandemic in resource-limited settings. However, the clinical course and outcomes of patients with COVID-19 admitted to this unique type of facility have never been studied. We retrospectively reviewed the medical records of adult patients with COVID-19 who were admitted to a single hospitel in Bangkok, Thailand. Risk factors with respect to chest X-ray progression and clinical progression were analyzed using a logistic regression. A total of 514 patients were recruited, with a mean (standard deviation) age of 35.6 (13.4) years, and 58.6% were women. Patients were admitted after a median (interquartile range) of 3 (2–6) days of illness and were classified with mild (12.3%), moderate (86.6%), and severe (1.1%) conditions. Favipiravir and corticosteroids were prescribed in 26.3% and 14.9% of patients, respectively. Chest X-ray progression was found in 7.6% of patients, and hospital transfer occurred in 2.9%, with no deaths. Favipiravir use (odds ratio (OR) 3.3, 95% confidence interval (CI) 1.4–7.5, *p* = 0.005), nausea/vomiting after admission (OR 32.3, 95% CI 1.5–700.8, *p* = 0.03), and higher oxygen saturation on admission (OR 1.99; 95% CI 1.22–3.23, *p* = 0.005) were factors associated with chest X-ray progression. Additionally, an oxygen requirement on admission was an independent risk factor for hospital transfer (OR 904, 95% CI 113–7242, *p* < 0.001). In a setting where the hospitel has been proposed as an extension facility for patients with relatively non-severe COVID-19, most patients could achieve a favorable clinical outcome. However, patients who require oxygen supplementation should be closely monitored for disease progression and promptly transferred to a hospital if necessary.

## 1. Introduction

Coronavirus disease 2019 (COVID-19) is an emerging infectious disease (ID) caused by severe acute respiratory syndrome coronavirus 2 (SARS-CoV-2), which has led to a global pandemic that is ongoing [1]. COVID-19 can cause respiratory system infection, with presentations varying from asymptomatic or mild upper respiratory tract disease to severe pneumonia. Individuals who develop respiratory failure requiring mechanical ventilation support have a risk of substantial morbidities and mortality [2]. Surging numbers of COVID-19 cases during an outbreak have challenged the health care systems in all countries worldwide, including in Thailand. When health care resources are insufficient to care for the number of patients, unfavorable consequences can occur, especially in settings where most individuals remain unimmunized against COVID-19 and vulnerable to infection [3,4].

With an increasing number of people with COVID-19 infection during the third wave of the pandemic in Thailand, the health care system reached its capacity. Therefore, home isolation with telemedicine service has been established to relieve the congestion of need and provide the most practical management for our patients. The goal is to monitor the patient closely and early on detect those at risk or who have severe COVID-19 and assist them to the facility if needed [5,6]. These similar services have been utilized in several countries [7,8,9]. However, based on the guideline established by the Ministry of Public Health of Thailand, all infected patients are required to be admitted to a healthcare facility for care and the prevention of transmission. Furthermore, with the new infection and the lack of knowledge, patients may be reluctant to remain in home isolation for this potentially severe disease during a pandemic.

A hospitel is a hotel designated as an extended healthcare facility for the patients to be admitted. Telemedicine care and non-parenteral medication were additionally offered to patients in the hospitel. Patients considered to have mild to moderate conditions or who lacked comorbidities were admitted to a hospitel to reduce the burden on regular hospitals [10]. Therefore, a hospitel could offer them a better approach, including oxygen supplementation, oral medication such as corticosteroids, and chest X-rays. The above reasons led to establishing an extended facility to assist in meeting the country’s urgent need for health care support [11]. However, the clinical course and outcomes of patients with COVID-19 at a hospitel in Thailand have never been thoroughly assessed. Therefore, we aimed to investigate these entities to provide a new perspective regarding auxiliary health care facilities during the COVID-19 pandemic. Our findings could lead to improved health care support in resource-constrained settings with a high number of patients requiring care and support. 

## 2. Materials and Methods

We retrospectively reviewed the medical records of all patients who were admitted to a hospitel in Bangkok, Thailand, between 26 April and 27 May 2021. The inclusion criteria were age ≥ 15 years and being directly admitted from home with a laboratory-confirmed diagnosis via the detection of SARS-CoV-2 using reverse transcription polymerase chain reaction (RT-PCR) (Bio-Rad Laboratories, Inc.) with nasopharyngeal swabs. Those who were readmitted or referred from other hospitels or other healthcare facilities were excluded. 

We aimed to investigate the clinical presentation and radiographic findings of patients with COVID-19 who were admitted to the hospitel. We collected patient data, including demographic, clinical, and radiological characteristics; details of treatment; and outcomes. The value for days of illness (DOI) was counted from the day of initial symptoms or the day of SARS-CoV-2 detection using RT-PCR in asymptomatic cases.

### 2.1. COVID-19 Management at the Hospitel

In mid-April 2021, the Faculty of Medicine Ramathibodi Hospital, Mahidol University, in collaboration with partner hotels, established numerous alternative health care facilities, referred to as the “Ramathibodi hospitels,” under a policy of the Ministry of Public Health of Thailand (MoPH) [12]. A hospitel is a hotel where patients are admitted to a single- or double-bed room. Physicians located in the hospitel lobby manage patients via telemedicine.

At our study hospitel, all patients were provided a thermometer and pulse oximeter upon admission, along with supportive medications such as paracetamol, antihistamines, and antitussive drugs. Each patient’s history of illness was taken over the phone without direct physical examination. Chest X-rays were conducted using a mobile or portable X-ray unit on admission (DOI 0–3) and on DOI 5–7 and DOI 10–12 or if clinically indicated. Patients were instructed to record their vital signs at least one time per day and report these to health care staff by sending a message via a secured online application. However, patients could contact our team 24 h a day if necessary via direct phone call. Physicians and health care staff working in the hospitels were voluntarily allocated from different departments under close supervision of ID physicians. These health care professionals made daily phone calls to obtain information regarding patients’ clinical symptoms and other possible complaints. 

Patient management guidelines were derived from the Department of Disease Control, MoPH, and verified by ID physicians [13]. Favipiravir was the only available antiviral medication for patients diagnosed with pneumonia initially or in the days following hospitel admission as well as for patients considered to have mild (with risk factors for severe disease) or moderate symptoms. Patients with peripheral capillary oxygen saturation (SpO_2_) < 95% were offered oxygen supplementation via a nasal cannula and dexamethasone. Under general conditions, intravenous medication and blood testing were not provided at the hospitel. Patients with a potential need for favipiravir, dexamethasone, or hospital referral were referred to an ID physician for determination. A patient with severe symptoms was referred to the hospital for further management and was either admitted to an airborne infection isolation room or the intensive care unit (ICU). According to the guidelines, all patients were discharged on DOI 14 or earlier but were required to isolate until DOI 14 or longer with an immunocompromised status. The dose administration of favipiravir (200 mg/tablet) was as follows: 1800 mg (nine tablets) twice daily on day 1 followed by 800 mg (four tablets) twice daily for 5 days. The duration was extended to 10 days in some patients whose symptoms did not improve by day 5. Patients with body weight > 90 kg received 2400 mg (12 tablets) twice daily on day 1 and 1000 mg (5 tablets) twice daily on the following days. Oral dexamethasone administration dosing was started from 6 mg once daily, with an increasing dose allowed if necessary [13]. All medications were provided to the patient on the first day of each regimen. A pharmacist was available to assist with the drug schedule and possible adverse reactions. 

Outcomes were focused on chest X-ray improvement or stability and disposition status from the hospitel. Patients transferred to a hospital were assessed for the need for intensive care, endotracheal intubation, length of stay, final disposition, and mortality.

### 2.2. Case Severity

All patients with COVID-19 were classified as having mild, moderate, or severe conditions for practical management. Mild COVID-19 was defined as the following: age < 60 years, no symptoms, no risk of severe diseases, SpO_2_ ≥ 96%, and no drop in SpO_2_ ≥ 3% after exercise. Patients with a risk of severe conditions, dyspnea, or respiratory rate > 20/min, SpO_2_ ≥ 96%, and no drop in SpO_2_ ≥ 3% after exercise were defined as having moderate illness. Patients with SpO_2_ < 96% or a drop in SpO_2_ ≥ 3% after exercise were classified as having severe illness. Risk factors for severe disease were defined according to the MoPH of Thailand and included underlying diabetes mellitus, cardiovascular disease, cerebrovascular disease, cancer, chronic kidney diseases, liver diseases, lung diseases, immunocompromised status, body weight > 90 kg, or body mass index (BMI) > 30 kg/m^2^ [13].

### 2.3. Radiographic Assessment

Rama Co-RADS is a tool used as an assessment scheme for chest X-ray findings to diagnose and monitor patients with COVID-19 pneumonia. Rama Co-RADS has been proposed and utilized throughout our institution (Table S1) [14]. This tool facilitates concise and objective communication between radiologists and physicians regarding the presence and changes of pulmonary abnormalities along with the clinical presentation. The criteria for improvement in chest X-ray or stability were determined for patients who had at least two chest X-rays performed at different time points. The definitions used were as follows: (i) chest X-ray remained within the same category; (ii) chest X-ray changed within category 1, 2, and C; and (iii) improvement on repeated chest X-ray to at least one category lower (e.g., from 5 to 4 or from 4 to 3) if prior chest X-ray was initially graded as category 3–5. 

### 2.4. Statistical Analysis

The means and standard deviations (SD) for absolute values and median values with interquartile ranges (IQR) for continuous variables are presented for patients’ demographic data, clinical and radiographic findings, and outcomes. The chi-square test or Fisher’s exact test and Wilcoxon rank-sum test were used to compare the data, as appropriate. An analysis of variance was used to compare the data across illness severity groups. A logistic regression analysis was used to assess factors associated with disease progression and outcomes, which are reported as odds ratios (OR) and 95% confidence intervals (CI). Those parameters with *p* < 0.05 in the univariate analysis were included in the multivariate analysis using forward stepwise selection. A value of *p* < 0.05 was considered statistically significant. The analysis was performed using Stata statistical software version 16 (StataCorp LLC, College Station, TX, USA). 

## 3. Results

**Figure 1:**
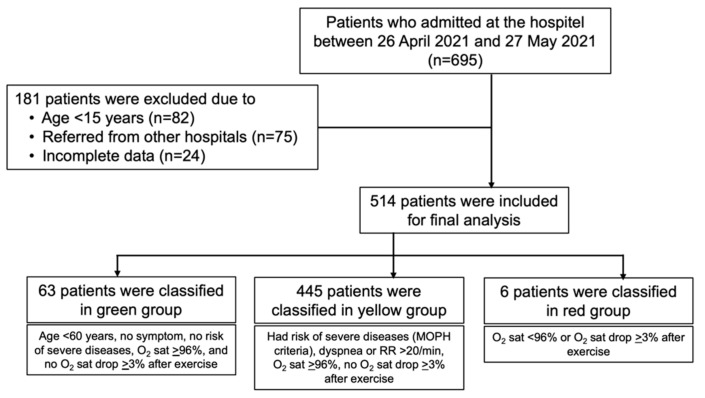
Study flow.

We retrieved data for 695 patients during the study period, and 514 patients were eventually included in the analysis (Figure 1). Demographic data of all patients are presented in Table 1. The mean (SD) patient age was 35.6 (13.4) years, and 58.6% were women. Among the evaluable patients, 63 (12.3%), 445 (86.6%), and 6 (1.2%) patients were initially classified as having mild, moderate, and severe conditions, respectively. Among them, 23.5% had underlying conditions, and 46.1% had a risk of severe disease. Patients were admitted after a median (IQR) of 3 (2–6) DOI. The most common presenting symptoms were cough (45.9%), fever (31.5%), sore throat (28.5%), nasal congestion (17.3%), and headache (10.9%). Among the 290 patients with available chest X-rays taken on DOI 1–3, the findings were unremarkable in 72.7%. Thirteen (2.5%) patients required oxygen supplementation. Approximately 26.3% received favipiravir for approximately 6 days, and 15% received oral dexamethasone for a duration of approximately 5 days.

Patients were classified as having mild (12.3%), moderate (86.6%), and severe (1.1%) illness severity. The proportions of men, patients with underlying diseases, patients with a risk of severe diseases, and patients with symptoms on admission were significantly different among groups (all *p* < 0.05) (Table 1). Additionally, the mean age, BMI, duration from initial symptoms until admission, respiratory rate, and SpO_2_ were also significantly different among groups (all *p* < 0.05). Patients with severe illness tended to be older and to have more underlying diseases, higher BMI, more symptoms, delayed admission, tachypnea, and relatively lower SpO_2_. Patients with severe conditions were more likely to have fever, cough, myalgia, and dyspnea. In contrast, those with moderate conditions were more likely to have a sore throat, diarrhea, anosmia, headache, nasal congestion, and sputum production. However, the mean body temperature and the category of chest X-ray findings on admission were not significantly different among the three conditions. 

**Figure 2:**
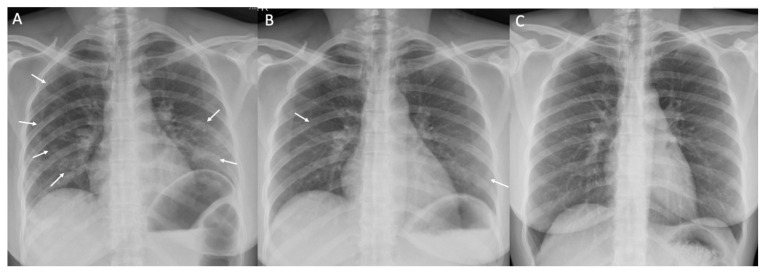
Chest X-rays during the hospitel course determined by Rama Co-RADS in one representative patient. A 43-year-old woman with a body mass index of 24 kg/m^2^ presented with cough and dyspnea for 1 day (13 days after contact). (**A**) Initial chest X-ray obtained on admission (day 2 of illness) shows multifocal poorly defined patchy opacities and/or consolidation (arrows) in both lungs, compatible with COVID-19 pneumonia (Rama Co-RADS category 5). (**B**) Follow-up chest X-ray obtained on day 6 of illness and after 4 days of favipiravir and corticosteroid treatment shows a marked improvement with faint residual opacities (arrows) in some affected areas. (**C**) Follow-up chest X-ray obtained on day 13 of illness shows complete resolution of COVID-19 pneumonia.

A total of 419 patients had at least two chest X-rays during admission to the hospitel. Thirty-two (8.8%) patients showed progression in their chest X-ray. However, 382 (91.2%) patients had improved or had stable radiographic findings. The latter group was compatible with criteria 1, 2, and 3 as follows: in total, 22 (5.8%), 2 (0.5%), and 138 (36.1%) patients were in the mild (73.4%), moderate (73.5%), and severe (33.3%) illness groups, respectively, with no significant difference in the proportions. One representative patient and set of chest X-rays during the hospitel course determined by Rama Co-RADS is presented in Figure 2. The factors associated with chest X-ray progression in multivariate logistic regression were oxygen saturation on admission (OR 1.99, 95% CI 1.23–3.23; *p* = 0.005), nausea/vomiting after admission (OR 32.33, 95% CI 1.49–700.82; *p* = 0.027), and administration of favipiravir (OR 3.27, 95% CI 1.43–7.50; *p* = 0.005).

For treatment outcomes (Table 2), most (n = 499) patients were discharged home from the hospitel with no events (97.1%), with a mean length of stay in the hospitel of approximately 10 days. A total of 15 (2.9%) patients were transferred to the hospital. Of those, a large proportion of patients in the severe condition group (50%) were referred out. Of the total, 2.2% were transferred to the general ward and 0.6% were transferred to the intermediate care unit. Only one (0.2%) patient required endotracheal intubation for respiratory support and was therefore admitted to a critical care unit. Oxygen saturation on admission was the only parameter that remained significantly associated with hospital transfer in the multivariate logistic regression analysis (OR 904, 95% CI 112.84–7242.55; *p* < 0.001). Nearly all patients eventually returned home (99.8%). However, only one patient remained admitted to the ICU during the study period. There were no deaths reported in our cohort.

## 4. Discussion

Herein, we describe an extended health care facility, the hospitel, which was used when there was a surge in the number of cases during the COVID-19 pandemic in Bangkok, Thailand. A hospitel can help to decrease the burden on hospitals, especially to care for patients with mild or moderate symptoms and when intravenous medication and routine blood work are not needed. The overall survival rate among patients treated in a hospitel is favorable, with only a few patients admitted to the ICU and no mortality observed. The use of a radiographic scale score is practical in real-world situations, mainly when direct examination is limited. We found that a higher SpO_2_, nausea/vomiting after admission, and receiving favipiravir could predict the progression of COVID-19 illness. Furthermore, patients with initially greater SpO_2_ on admission were more likely to be transferred to the hospital, regardless of other adjusted covariables.

The COVID-19 pandemic has challenged health care systems in all countries due to a high demand for health services, which has overwhelmed health care facilities and medical personnel. Therefore, extended health care facilities such as a field hospital or hospitel have been established to facilitate the provision of advanced care for patients who require less intensive monitoring. These auxiliary facilities have been used during epidemic waves of COVID-19 in several countries; however, the effectiveness of these unique facilities, especially the hospitel, has rarely been reported. We found that patients with mild to moderate symptoms could conduct self-monitoring together with close supervision by clinicians via telemedicine. Telemedicine is an emerging tool that is practical in assisting clinicians during the COVID-19 pandemic. We observed a potential utilization of this strategy, especially when the disease is considered non-severe and monitoring equipment is available. In addition, telemedicine could reduce exposure to infected patients and subsequent disease acquisition. Furthermore, there is an opportunity to implement telemedicine for those with effective medication and stable conditions in the near future [15,16]. However, although classification is a practical tool that could assist clinicians to triage patients initially, the discordance in symptoms and clinical findings can sometimes limit its use, and some patients can be misclassified. Oral antiviral agents, corticosteroids, and supportive therapy are essential for a favorable patient outcome. Although the efficacy of favipiravir to prevent disease progression remains uncertain, this drug has been used in settings where remdesivir or other immunomodulatory agents are out of reach. Recently published data in Thailand have revealed only mild intolerance related to favipiravir, without severe adverse reactions [17].

Pulmonary examination for all patients during a pandemic is limited due to the need for universal protection for physicians and the low sensitivity to detect the early or very mild stages of COVID-19. Therefore, a chest X-ray assessment scheme that is practical and accessible for every facility has shown potential usefulness in the diagnosis, monitoring, and prediction of patients with a risk of severe disease. Our Rama Co-RADS was used to support chest X-ray examination and has the potential to assist in the telemedicine monitoring of disease progression and outcomes. The Rama Co-RADS scale is practical and easy to follow. A similar chest X-ray assessment scheme platform that uses a score to grade severity has been described in the literature to provide the frequency and distribution of chest X-ray findings and their association with outcomes among patients with COVID-19. That scale has been prognostically used in a resource-constrained setting to predict patients at risk of ICU admission and mortality [18]. 

There was no clear explanation for why patients with higher peripheral capillary SpO_2_ on admission had a greater likelihood of chest X-ray progression and hospital transfer. This could be explained by a better baseline value of SpO_2_, which can be used to monitor disease progression as compared with patients who have low SpO_2_ and would be more likely to be referred initially. Furthermore, patients with nausea/vomiting after admission could have impaired gastrointestinal absorption, which would result in a subtherapeutic levels of administered medications. However, nausea/vomiting after admission might predict COVID-19 disease progression. Additionally, we found that patients who received oral favipiravir tended to have more severe COVID-19 illness.

In terms of outcome evaluation, only a few patients were transferred to the hospital, and a small number of patients required admission to the ICU. There were no deaths among our patients. Early intervention could explain this, with good monitoring in patients with mild COVID-19 illness. Our study was conducted when the rate of immunization in Thailand had not yet reached 70% of the population. We also observed that a hospitel might be more suitable for patients, especially when home isolation is not possible, such as in a large extended family with several members living under the same roof. 

This study has some limitations. First, recall bias could not be avoided. Second, the severity criteria used in this study may not be universally compatible. Furthermore, we encourage evaluating the patients individually rather than relying on only single parameters such as SpO_2_. Third, there were missing chest X-rays and limited data on the ultimate outcomes of some patients after transfer to a hospital. Fourth, regarding antiviral agents, our patients received corticosteroids approved for patients hospitalized with COVID-19 [19]; the use of dexamethasone resulted in lower 28-day mortality among those who were receiving either invasive mechanical ventilation or oxygen [20]. Last, favipiravir could be a cofounder of disease progression, and it may not be possible to determine whether this is an actual risk factor. However, the strengths of this study include that we first investigated a unique type of facility for patients with COVID-19, and our study included a large number of patients. This type of supportive facility is practical and logical for use when needed during a pandemic. Additionally, the use of Rama Co-RADS, the newly proposed categorical scheme for chest X-ray findings, enabled us to monitor the progression of the disease efficiently and avoid subjective measurement.

## 5. Conclusions

An extension healthcare facility such as a hospitel was deemed practical for treating patients with relatively non-severe COVID-19 during the pandemic in areas where local health care facilities are overwhelmed due to a large increase in the number of patients. Most patients can achieve a favorable clinical outcome with telemonitoring and radiographic assessment tools. In our study, patients who developed nausea/vomiting at admission and received favipiravir were independently associated with chest X-ray progression; these patients may need to be monitored closely. Patients who initially had high oxygen saturation should also be closely monitored for disease progression and promptly transferred to a hospital if necessary.

## Figures and Tables

**Table 1:** Baseline characteristics and clinical presentation of 514 patients.

Variables	Total (n = 514)	Mild (n = 63)	Moderate (n = 445)	Severe (n = 6)	*p*-Value
Clinical characteristics					
Male sex, n (%)	213 (41.4)	35 (55.6)	175 (39.3)	3 (50)	0.038
Age (years), mean (SD)	35.6 (13.4)	34.4 (11.5)	35.5 (13.6)	51.7 (13)	0.010
Age > 60 years, n (%)	28 (5.4)	0 (0)	27 (6.1)	1 (16.7)	0.285
Underlying diseases, n (%)	121 (23.5)	11 (17.5)	106 (23.8)	4 (66.7)	0.029
BMI (kg/m^2^), mean (SD)	23.6 (4.0)	22.7 (3.1)	23.7 (4.1)	28.2 (5.4)	0.022
Risk of severe disease, n (%) *	237 (46.1)	0	232 (52.1)	5 (83.3)	<0.001
Symptomatic before admission, n (%)	388 (75.5)	0	382 (85.8)	6 (100)	<0.001
Time from symptom or positive PCR to admission (days), median (IQR)	3 (2–6)	1 (1–2)	4 (2–6)	8.5 (5–11)	<0.001
Clinical presentation					
Fever, n (%)	162 (31.5)	1 (1.6)	158 (35.5)	3 (50.0)	<0.001
Sore throat, n (%)	146 (28.4)	2 (3.2)	143 (32.1)	1 (16.7)	<0.001
Cough, n (%)	236 (45.9)	2 (3.2)	230 (51.7)	4 (66.7)	<0.001
Diarrhea, n (%)	22 (4.3)	0	22 (4.9)	0	0.199
Anosmia, n (%)	41 (8.0)	0	41 (9.2)	0	0.019
Myalgia, n (%)	50 (9.7)	0	49 (11.0)	1 (16.7)	0.003
Dyspnea, n (%)	32 (6.2)	0	29 (6.5)	3 (50.0)	<0.001
Headache, n (%)	56 (10.9)	0	56 (12.6)	0	0.002
Nasal congestion, n (%)	89 (17.3)	2 (3.2)	87 (19.6)	0	0.001
Red eyes, n (%)	3 (0.6)	0	3 (0.67)	0	1.000
Ageusia, n (%)	17 (3.3)	0	17 (3.82)	0	0.387
Sputum production, n (%)	35 (6.8)	0	35 (7.9)	0	0.030
Nausea/vomiting, n (%)	4 (0.8)	0	4 (0.9)	0	1.000
Chest pain, n (%)	21 (4.1)	0	20 (4.5)	1 (16.7)	0.060
Fatigue, n (%)	8 (1.6)	0	8 (1.8)	0	0.640
Other symptoms, n (%)	15 (2.2)	0	15 (3.4)	0	0.361
Physical examination and chest X-ray findings					
Body temperature (°C), mean (SD)	36.6 (0.7)	36.7 (0.6)	36.6 (0.7)	36.8 (1.2)	0.404
Respiratory rate (breaths/min), mean (SD)	20 (1)	20 (1.0)	20 (1.0)	23 (6)	<0.001
SpO_2_ at rest (%), mean (SD)	98.0 (1.0)	98.2 (0.8)	98.0 (1.0)	96 (3.5)	<0.001
Normal chest X-ray on DOI 1–3, n (%)	211/290 (72.7)	47/57 (82.5)	163/231 (70.6)	1/2 (50)	0.136
Treatment					
Received oxygen supplementation, n (%)	13 (2.5)	0	10 (2.2)	3 (50)	<0.001
Received favipiravir, n (%)	135 (26.3)	9 (14.3)	120 (27.0)	6 (100)	<0.001
Duration of favipiravir (days), mean (SD)	5.7 (1.7)	5.9 (2)	5.6 (1.7)	7.5 (2.7)	0.042
Received corticosteroids, n (%)	77 (15.0)	3 (4.8)	68 (15.3)	6 (100.0)	<0.001
Duration of corticosteroids (days), mean (SD)	5.2 (2.0)	7 (1.7)	5 (1.9)	6.3 (2.6)	0.068

BMI, body mass index; IQR, interquartile range; PCR, polymerase chain reaction; SD, standard deviation; SpO_2_, oxygen saturation; DOI, date of illness; * underlying diabetes mellitus, cardiovascular disease, cerebrovascular disease, cancer, chronic kidney diseases, liver diseases, lung diseases, immunocompromised status, body weight > 90 kg, or body mass index (BMI) > 30 kg/m^2^ [13].

**Table 2:** Clinical outcomes of 514 patients.

Variables	Total (n = 514)	Mild (n = 63)	Moderate (n = 445)	Severe (n = 6)	*p*-Value
Chest X-ray improved/stable *, n (%)	387 (92.4)	58 (92.1)	327 (73.5)	2 (33.3)	0.660
Discharged home from the hospitel, n (%)	499 (97.1)	63 (100)	433 (97.3)	3 (50)	<0.001
Duration of hospitel admission (days), mean (SD)	9.4 (3.3)	11.8 (2)	9 (3.3)	9 (5.3)	<0.001
Transferred to general ward, n (%)	11 (2.2)	0	8 (1.8)	3 (50)	<0.001
Transferred to intermediate ward, n (%)	3 (0.6)	0	3 (0.7)	0	1.000
Transferred to ICU, n (%)	1 (0.2)	0	1 (0.2)	0	1.000
Endotracheal intubation, n (%)	1 (0.2)	0	1 (0.2)	0	1.000
Discharged home finally, n (%)	513 (99.8)	63 (100)	444 (99.8)	6 (100)	1.000
Duration of overall admission (days), mean (SD)	9.5 (3.5)	11.8 (2)	9.2 (3.5)	8.3 (1.2)	<0.001
Deaths, n (%)	0	0	0	0	N/A

* Among 419 evaluable patients. ICU, intensive care unit; SD, standard deviation.

## Data Availability

All relevant data are presented within the manuscript.

## Supplementary Materials

The following supporting information can be downloaded at: https://www.mdpi.com/article/10.3390/tropicalmed7090238/s1, Table S1: Rama Co-RADS: A tool used as an assessment scheme for chest X-ray findings to diagnose and monitor patients with COVID-19 pneumonia.

## References

[1] Azevedo T.C.P.D., Melo V.S.C., Silva R.M.D., Barbosa B.G.D.H., Christofoletti L.Z.D.M., Nascimento G.M.C.S.D., Oliveira G.S.L.D., Barbosa F.T., Sousa-Rodrigues C.F.D. (2021). Update of the epidemiological distribution of COVID-19 variants: A review article. Rev. Assoc. Med. Bras..

[2] Bruminhent J., Ruangsubvilai N., Nabhindhakara J., Ingsathit A., Kiertiburanakul S. (2020). Clinical characteristics and risk factors for coronavirus disease 2019 (COVID-19) among patients under investigation in Thailand. PLoS ONE.

[3] Emanuel E.J., Persad G., Upshur R., Thome B., Parker M., Glickman A., Zhang C., Boyle C., Smith M., Phillips J.P. (2020). Fair allocation of scarce medical resources in the time of COVID-19. N. Engl. J. Med..

[4] Lin C.A., Franco J.B., da Costa Ribeiro S.C., Dadalto L., Letaif L.S.H. (2021). Scarce resource allocation for critically ill patients during the COVID-19 pandemic: A public health mergency in São Paulo Brazil. Clinics.

[5] Elezkurtaj S., Greuel S., Ihlow J., Michaelis E.G., Bischoff P., Kunze C.A., Sinn B.V., Gerhold M., Hauptmann K., Ingold-Heppner B. (2021). Causes of death and comorbidities in hospitalized patients with COVID-19. Sci. Rep..

[6] Pinyopornpanish K., Nantsupawat N., Buawangpong N., Pliannuom S., Vaniyapong T., Jiraporncharoen W. (2022). Concerns of Home Isolating COVID-19 Patients While Receiving Care via Telemedicine during the Pandemic in the Northern Thailand: A Qualitative Study on Text Messaging. Int. J. Environ. Res. Public Health.

[7] Yılmaz Z., Duman S., Öztürk G., Özdemir H., Hogan G., Karataş K. (2021). Evaluating the home isolation of COVID-19 patients in primary care. J. Ideas Health.

[8] Wurzer D., Spielhagen P., Siegmann A., Gercekcioglu A., Gorgass J., Henze S., Kolar Y., Koneberg F., Kukkonen S., McGowan H. (2021). Remote monitoring of COVID-19 positive high-risk patients in domestic isolation: A feasibility study. PLoS ONE.

[9] Bhardwaj P., Joshi N.K., Gupta M.K., Goel A.D., Saurabh S., Charan J., Rajpurohit P., Ola S., Singh P., Bisht S. (2021). Analysis of Facility and Home Isolation Strategies in COVID 19 Pandemic: Evidences from Jodhpur, India. Infect. Drug Resist..

[10] Prutipinyo C. (2021). COVID-19 field hospital: Alternative state quarantine hospital and hospitel. Public Health Policy Laws J..

[11] Department of Disease Control MoPH (2021). Corona Virus Disease (COVID-19): Thailand Situation. https://ddc.moph.go.th/viralpneumonia/eng/index.php.

[12] Department of Disease Control MoPH (2021). Hospitel. http://www.hsscovid.com/img/helphospitel.pdf.

[13] Department of Disease Control MoPH (2021). Guidelines on Clinical Practice, Diagnosis, Treatment, and Prevention of Healthcare-Associated Infection for COVID-19. https://ddc.moph.go.th/viralpneumonia/eng/file/guidelines/g_CPG_28jan21.pdf.

[14] Suwatanapongched T., Nitiwarangkul C., Arnuntasupakul V., Kiertiburanakul S. (2021). Rama Co-RADS: Cutting-edge tool for improved communication in management and treatment of COVID-19 patients in Thailand. ASEAN J. Radiol..

[15] Pogorzelska K., Chlabicz S. (2022). Patient Satisfaction with Telemedicine during the COVID-19 Pandemic—A Systematic Review. Int. J. Environ. Res. Public Health.

[16] Davarpanah A.H., Mahdavi A., Sabri A., Langroudi T.F., Kahkouee S., Haseli S., Kazemi M.A., Mehrian P., Mahdavi A., Falahati F. (2020). Novel Screening and Triage Strategy in Iran During Deadly Coronavirus Disease 2019 (COVID-19) Epidemic: Value of Humanitarian Teleconsultation Service. J. Am. Coll. Radiol..

[17] Sirijatuphat R., Suputtamongkol Y., Angkasekwinai N., Horthongkham N., Chayakulkeeree M., Rattanaumpawan P., Koomanachai P., Assanasen S., Rongrungruang Y., Chierakul N. (2021). Epidemiology, clinical characteristics, and treatment outcomes of patients with COVID-19 at Thailand’s university-based referral hospital. BMC Infect. Dis..

[18] Kaleemi R., Hilal K., Arshad A., Martins R.S., Nankani A., Tu H., Basharat S., Ansar Z. (2021). The association of chest radiographic findings and severity scoring with clinical outcomes in patients with COVID-19 presenting to the emergency department of a tertiary care hospital in Pakistan. PLoS ONE.

[19] Chuah C.H., Chow T.S., Hor C.P., Cheng J.T., Ker H.B., Lee H.G., Lee K.S., Nordin N., Ng T.K., Zaid M. (2021). Efficacy of early treatment with favipiravir on disease progression among high risk COVID-19 patients: A randomized, open-label clinical trial. Clin. Infect. Dis..

[20] The RECOVERY Collaborative Group (2021). Dexamethasone in hospitalized patients with COVID-19. N. Engl. J. Med..

