# Supplementary material for: Clinical Course and Outcomes among COVID-19 Patients at the Hospitel in Bangkok: A Retrospective Study
Source: Trop Med Infect Dis. 2022 Sep 10;7(9):238. doi: 10.3390/tropicalmed7090238 (PMC9506055; doi:10.3390/tropicalmed7090238)
Supplement: Supplementary file 1 [file tropicalmed-07-00238-s001.zip › tropicalmed-1877172-supplementary.pdf]

Tropical Medicine and Infectious Disease

Supplementary Materials: Clinical Course and Outcomes among COVID-19 Patients at the Hospitel in Bangkok: A Retrospective Study

Table S1. Rama Co-RADS: A tool used as an assessment scheme for chest X-ray findings to diagnose and monitor patients with COVID-19 pneumonia.

Rama Co-RADS	Likelihood of COVID-19 Pneumonia	Impression	Chest X-ray Findings
**Category 1**	Negative or very low	Normal chest X-ray	No abnormality detected.
**Category 2**	Negative or very low	Minor abnormalities unrelated to COVID-19 pneumonia	Anatomical variants (e.g., breast implants, scoliosis, and old bony fractures)<br>Features favoring minor technical issues (e.g., suboptimal inspiration, inadequate exposure) but not affecting film interpretation<br>Irrelevant abnormalities (e.g., old tuberculosis, mild cardiomegaly, aortic atherosclerosis)
**Category C**	Low	Low probability or atypical for COVID-19 pneumonia, but with other clinically important diseases requiring clinical correlation and further management	Other clinically important diseases (e.g., bacterial pneumonia, active tuberculosis, congestive heart failure, pneumothorax, pleural effusion, malignancy)<br>Presence of severe technical artifacts affecting image interpretation and requiring repeated or follow-up chest X-ray
**Category 3**	Moderate	Equivocal/unsure/indeterminate for COVID-19 pneumonia	Some features (e.g., subtle, poorly defined opacities) that may represent early/mild/atypical COVID-19 pneumonia or other causes (e.g., pseudolesions, other diseases) requiring clinical correlation and follow-up chest X-ray
**Category 4**	High	Suspicious for early/mild COVID-19 pneumonia	Single or multifocal, poorly defined ground-glass opacities or consolidations in unilateral lung
**Category 5**	Very high	Typical for COVID-19 pneumonia	Multifocal peripheral opacities (ground-glass opacities and/or consolidations), including those with rounded morphology, in bilateral lungs

Adapted from Suwatanapongched et al., 2021 [14].
